# Supplementary material for: Changes in Rumen Microbiota Affect Metabolites, Immune Responses and Antioxidant Enzyme Activities of Sheep under Cold Stimulation
Source: Animals (Basel). 2021 Mar 5;11(3):712. doi: 10.3390/ani11030712 (PMC7999998; doi:10.3390/ani11030712)
Supplement: Supplementary file 1 [file animals-11-00712-s001.pdf]

animals

MDPI

Article

Changes in Rumen Microbiota Affect Metabolites, Immune Responses and Antioxidant Enzyme Activities of Sheep under Cold Stimulation

Hongran Guo †, Guangchen Zhou †, Guangjie Tian, Yuyang Liu, Ning Dong, Linfang Li, Shijun Zhang, Haochen Chai, Yulin Chen and Yuxin Yang *

Supplementary Material

Supplementary Material S1

All computational procedures were performed using the following packages available in R (3.5.0) software: Base R, nlem, psych, stats, corrplot, ggplot2 and agricolae. The normality of the distribution of residues and homogeneity of the variances in all data were tested using the Shapiro-Wilk and Bartlett tests ($P > 0.05$), respectively. Data were analyzed using the nlem package of R for a completely randomized design. A mixed model was constructed that included: windy velocity as fixed effects, and goat ID as random effects. The Duncan test of the agricolae package was used for multiple comparisons. The Duncan test of the agricolae package was used for multiple comparisons. The spearman of corrplot was used for correlation analysis. $P<0.05$ was considered statistically significant. The ggplot2 package was used to plot the figures.

Supplementary Table

Table S1. Composition and nutrient levels of the sheep diet (air-dry basis).

Feed Composition	Content (%)	Nutrient Level	Content （%）
Ingredient		DM	90.57
Corn	34	GE/ (MJ/kg)	16.6
Barley straw	30.5	CP	14,69
Sunflower cake	10	Ca	1.02
Corn fiber	4	Total P	0.42
Cottonseed meal	7.4	CF	14.78
Wheat middlings	4	EE	1.48
Malt sprouts	3.5	Ash	9.56
Soybean meal	2		
Limestone	1		
Molasses	1.5		
Urea	0.5		
$NaHCO_3$	0.5		
Salt	0.5		
Mineral premix	0.2		
CaH_2PO_4	0.32		
Vitamin premix	0.03		
Monensin (20%)	0.02		
Sweetening agent	0.02		
Flavouring agent	0.01		
Total	100		

Supplementary Figure

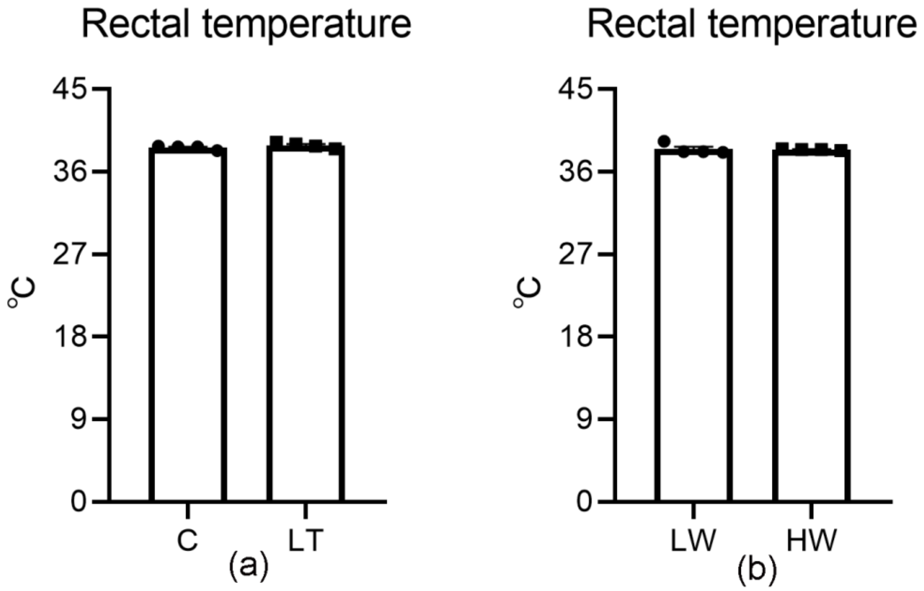

Figure S1. Changes in sheep rectal temperature during cold stress.
